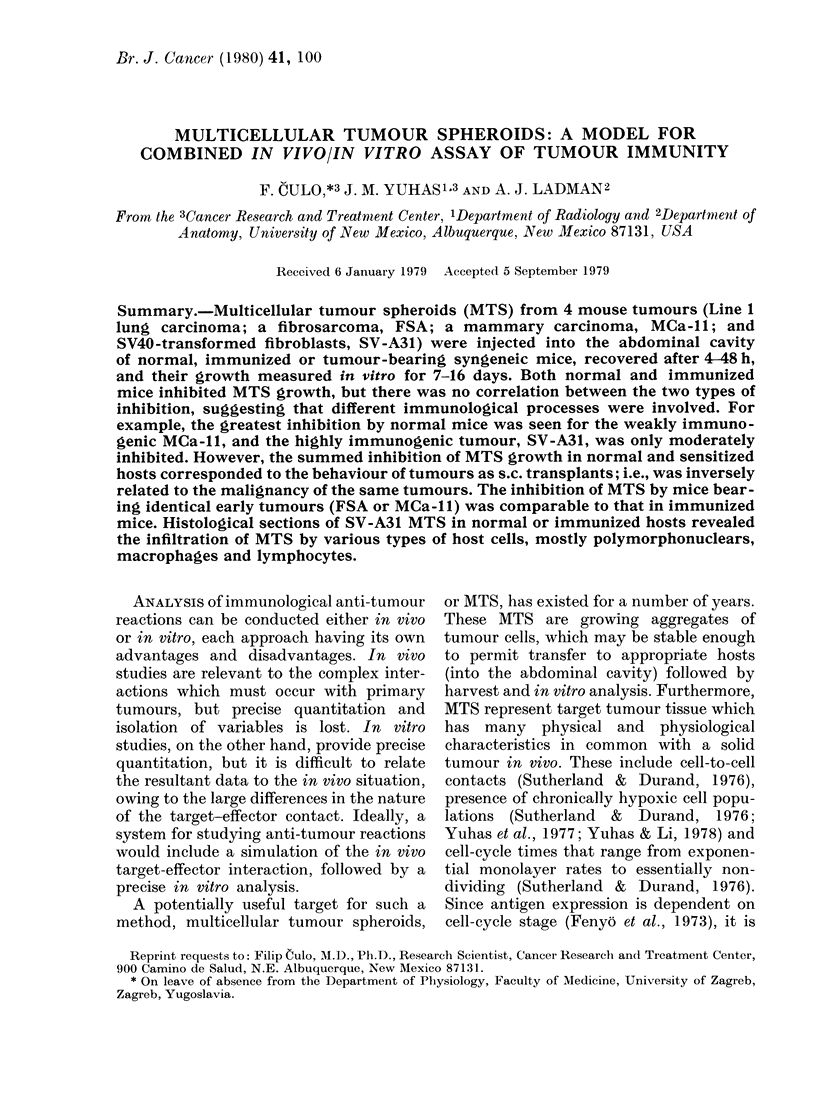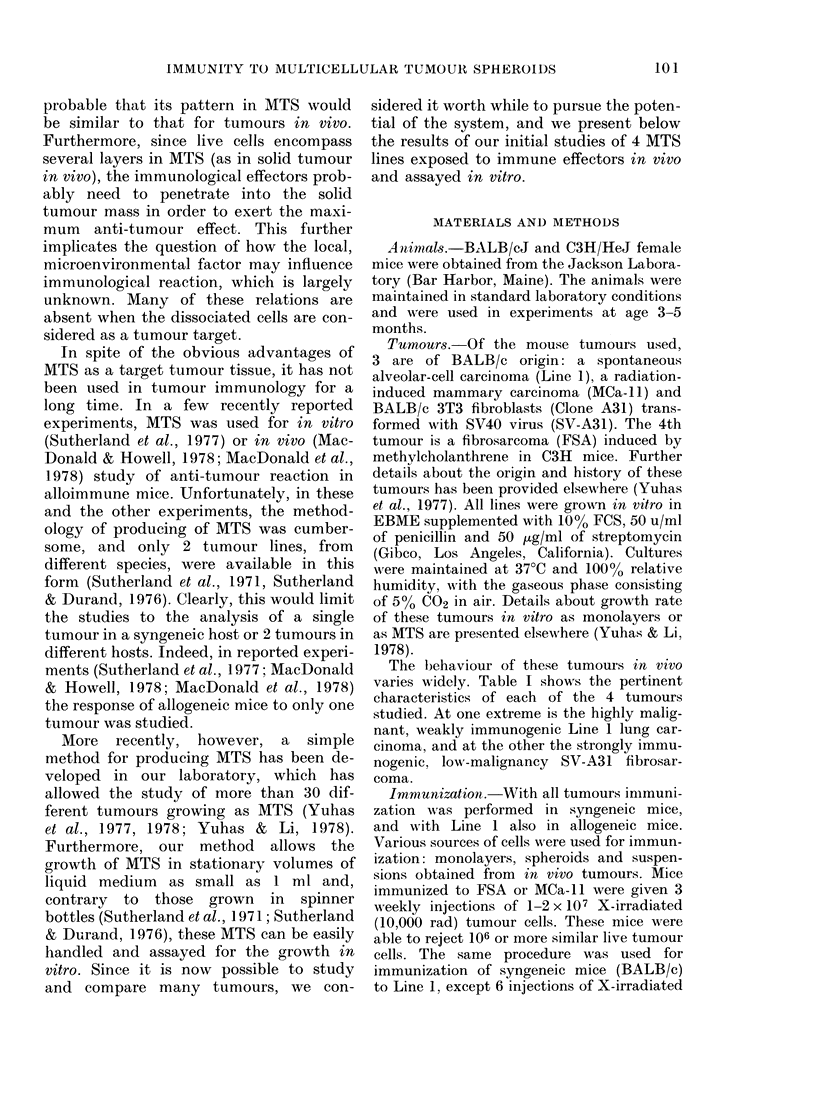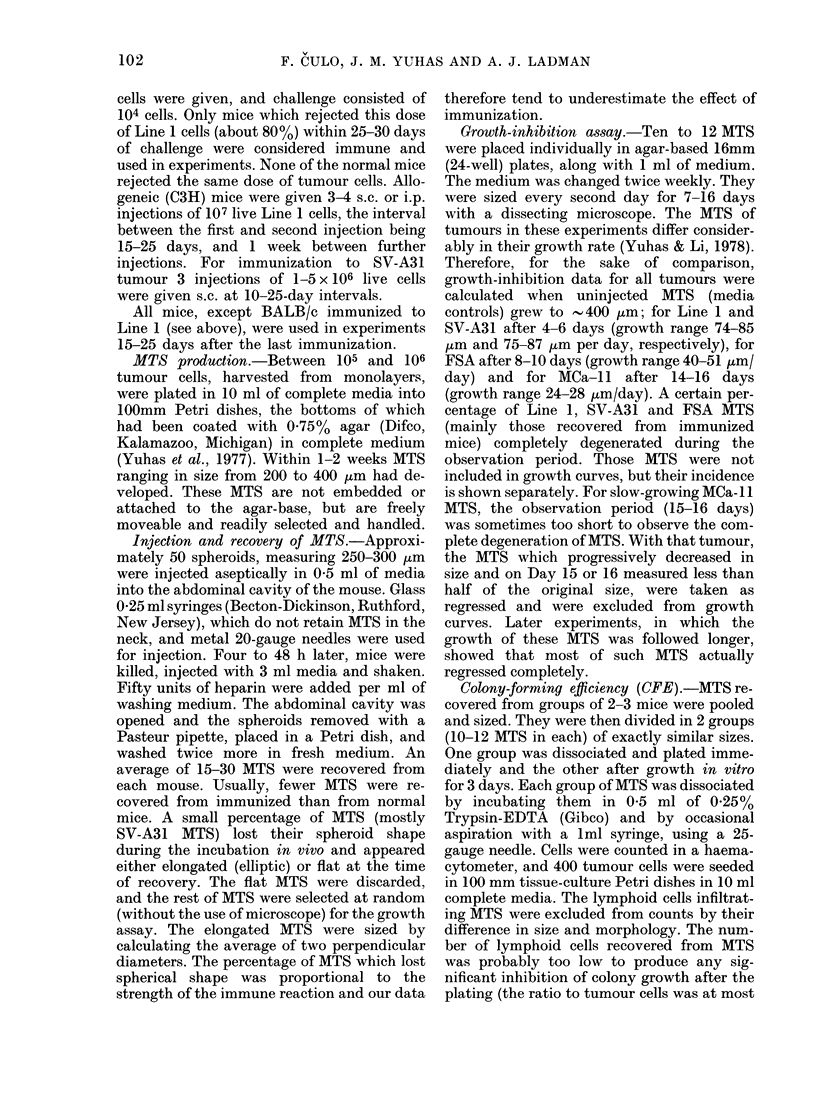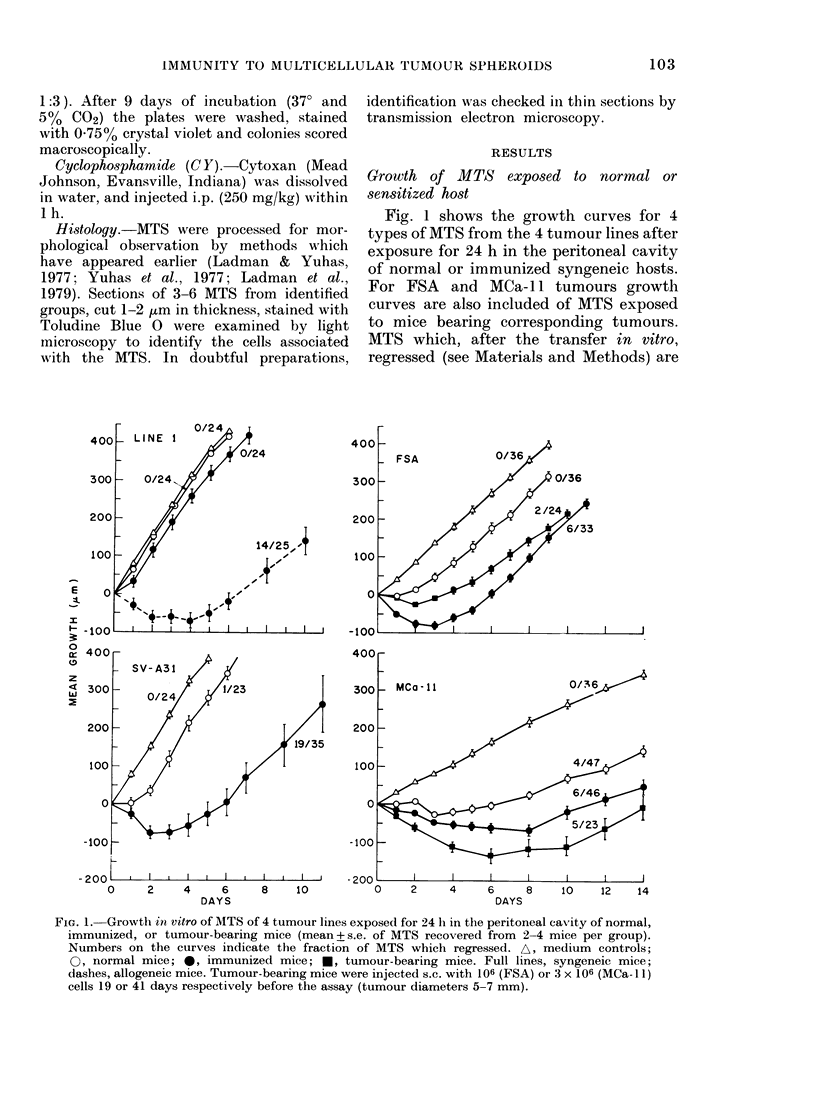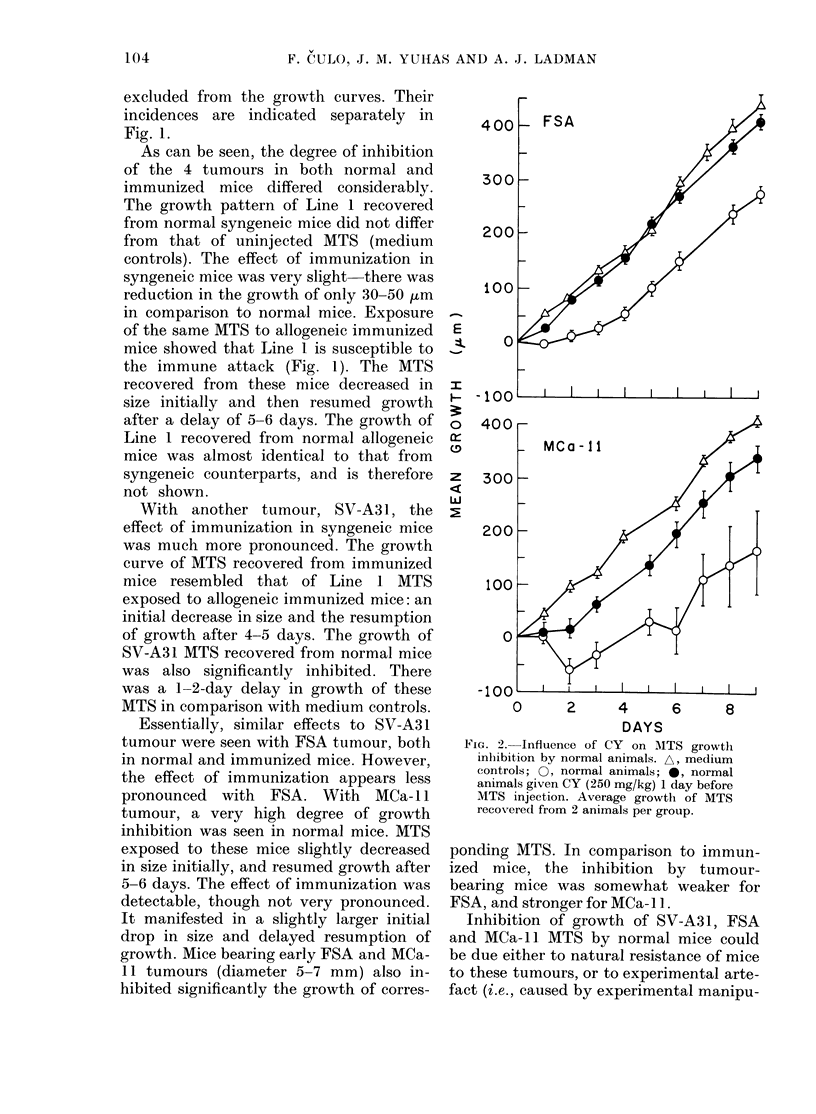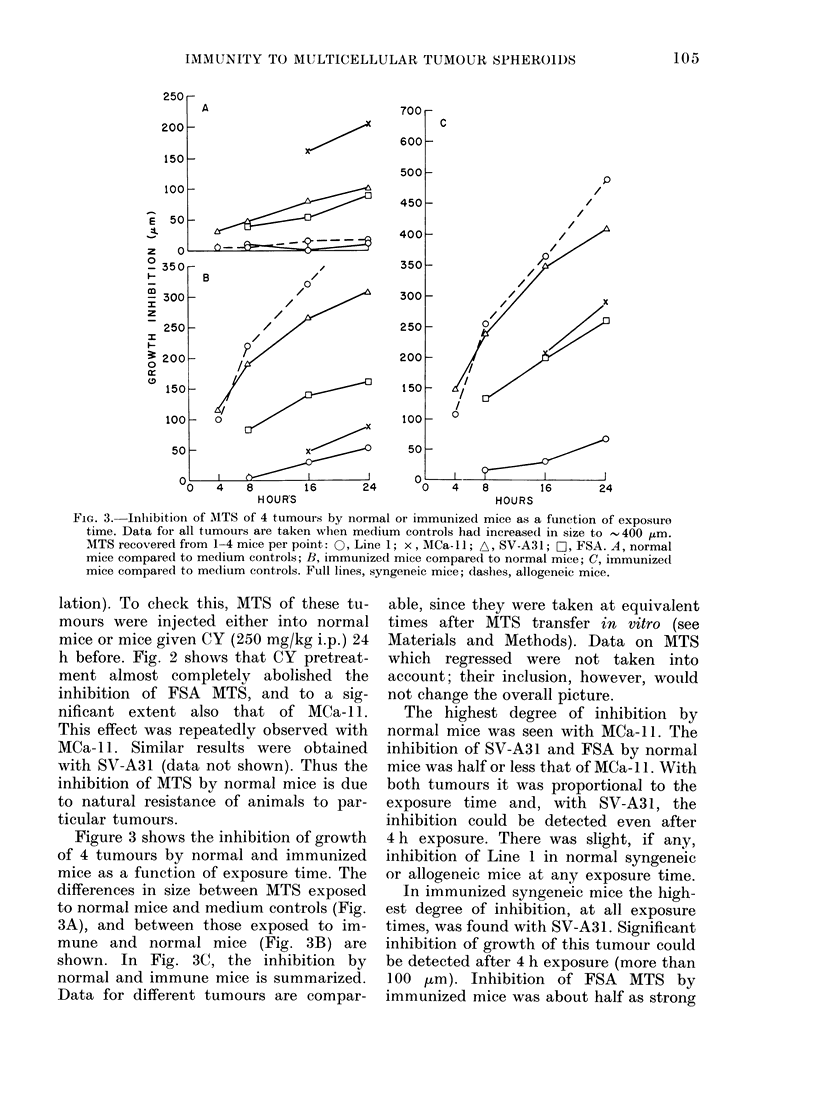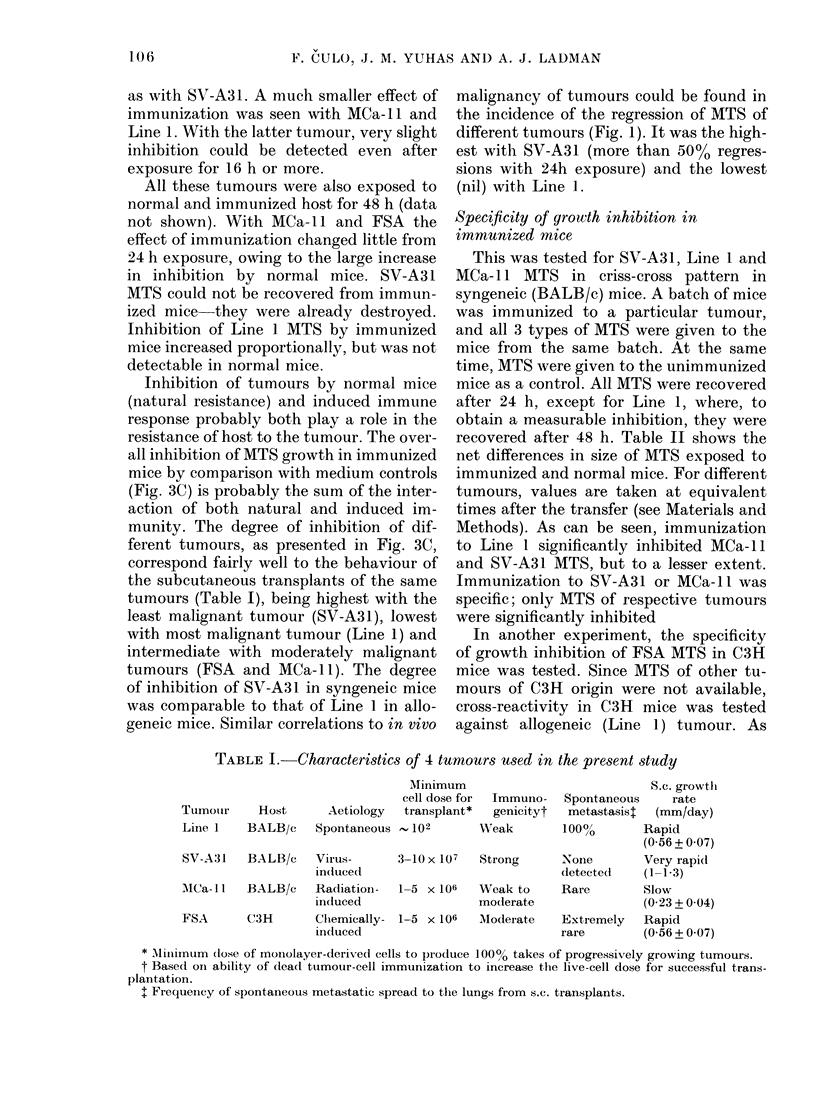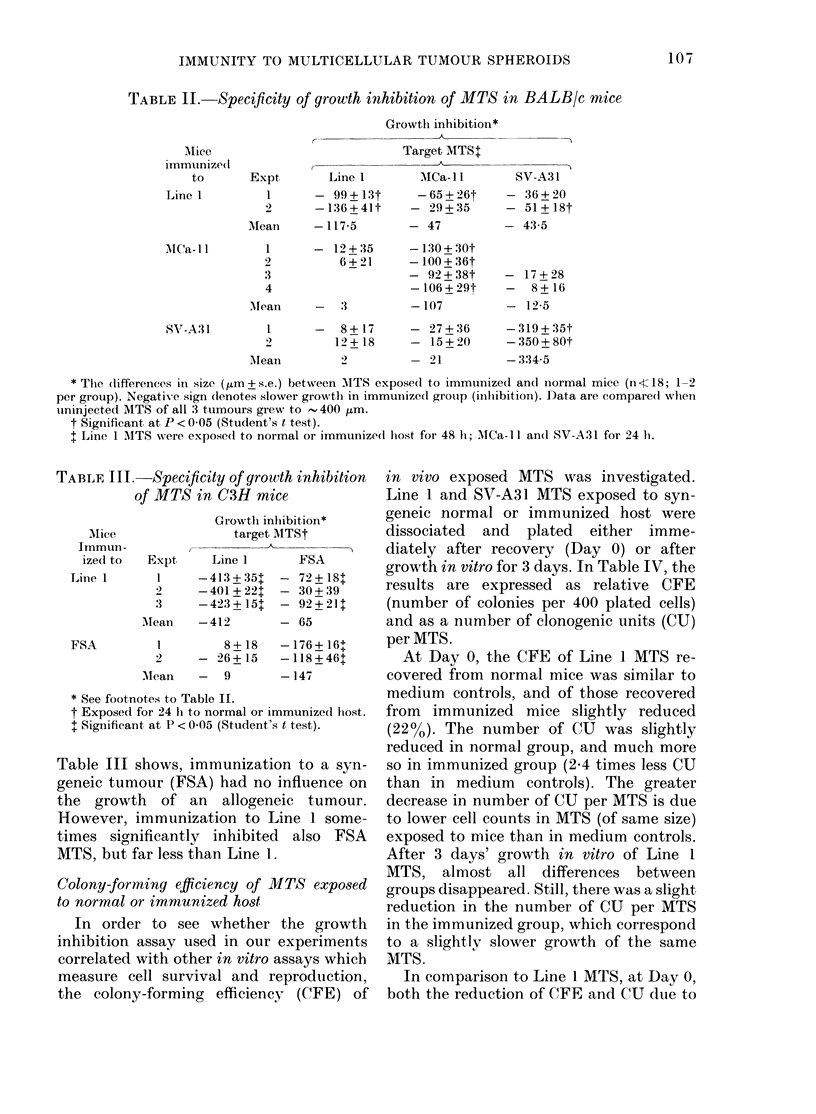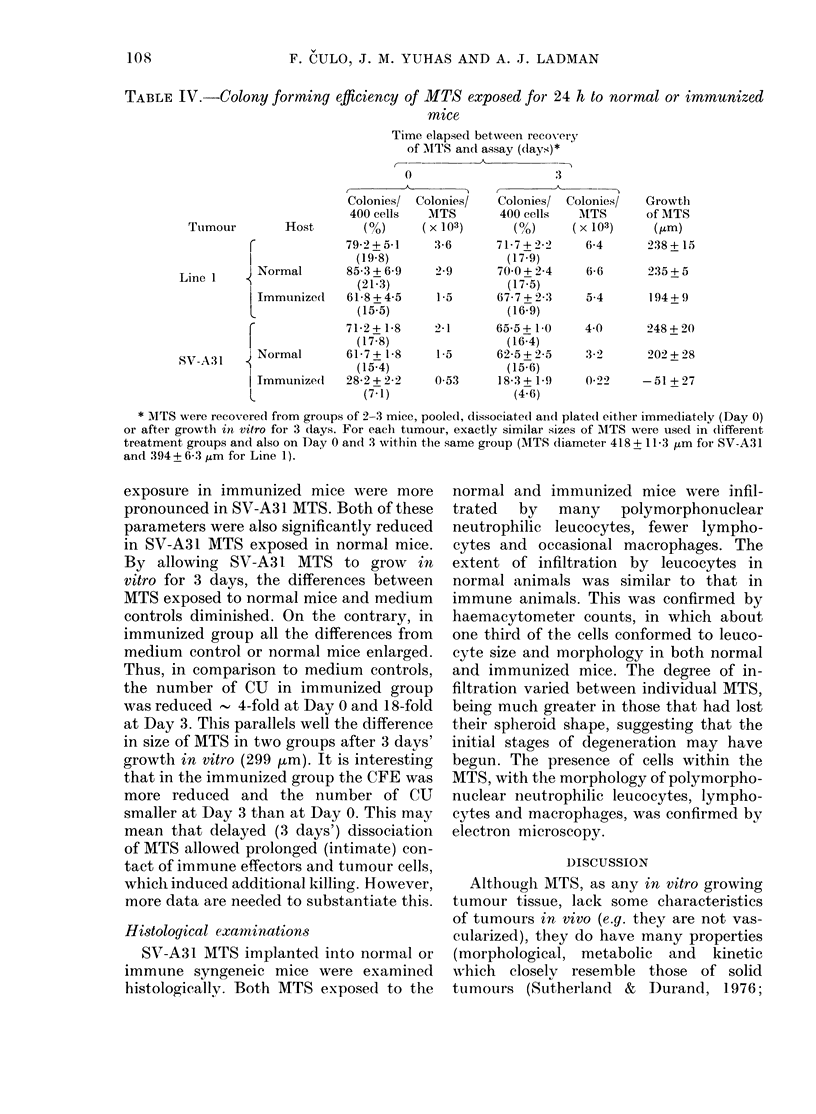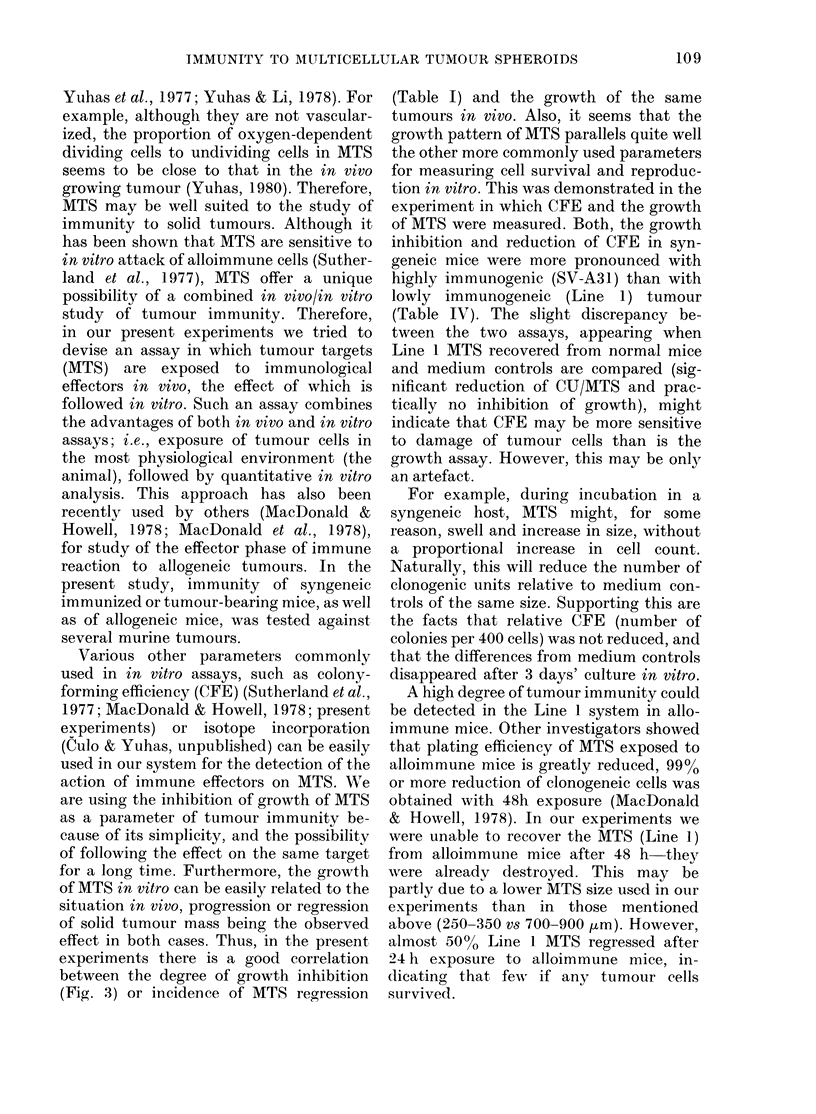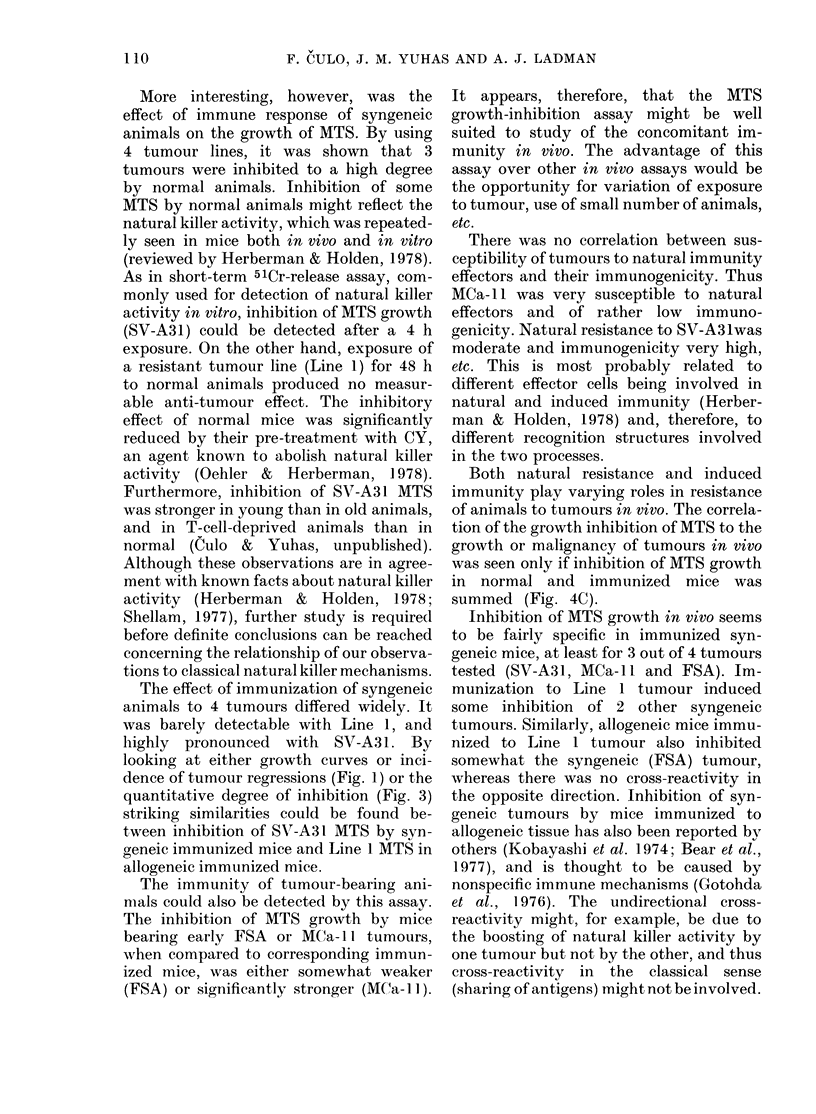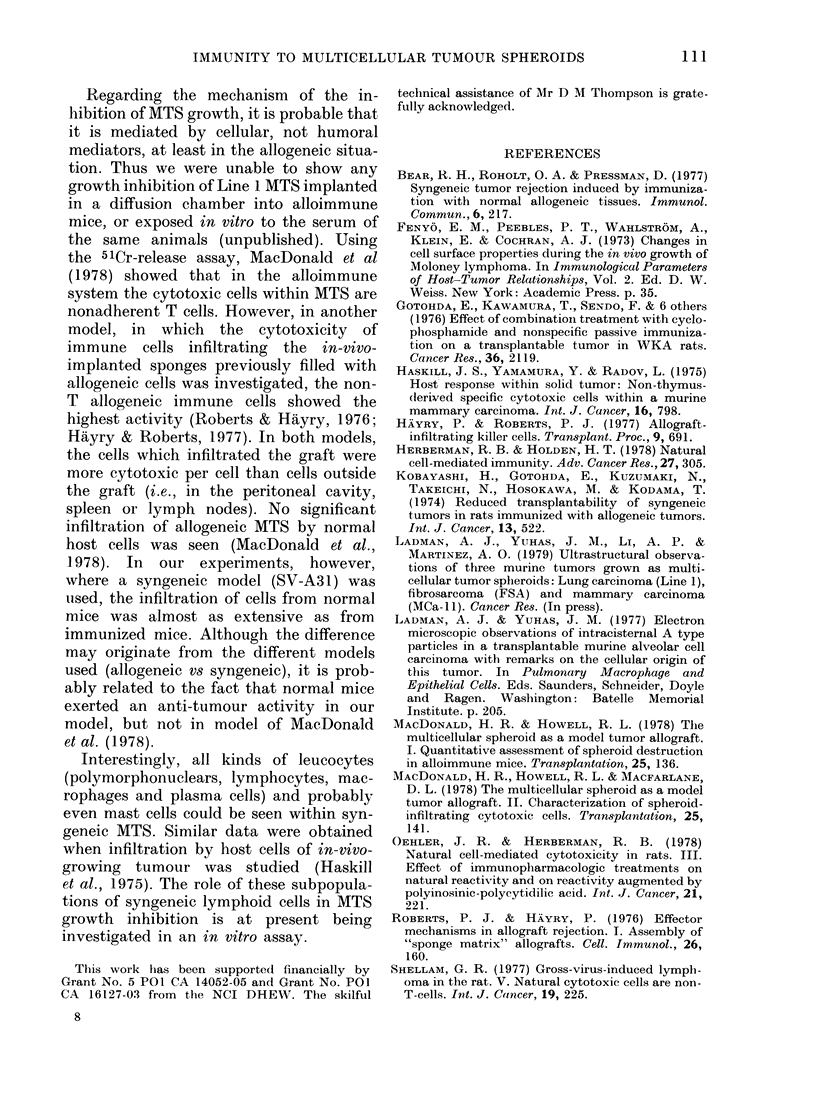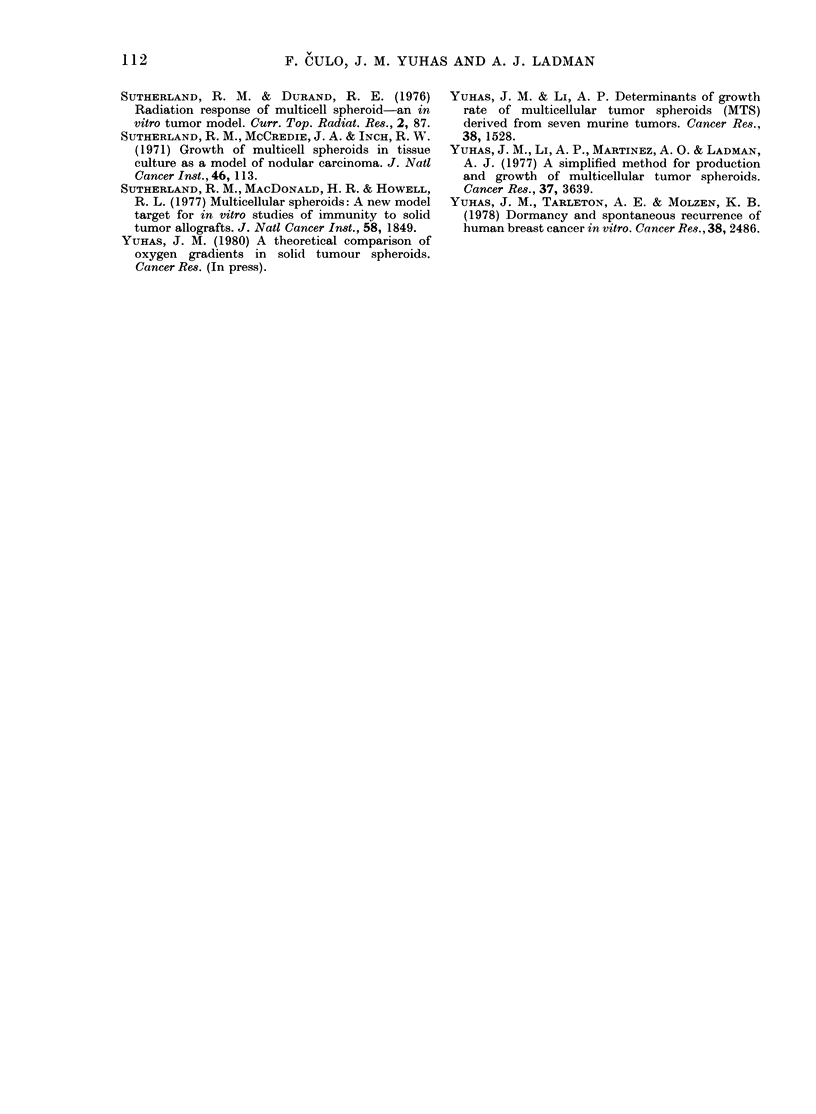# Multicellular tumour spheroids: a model for combined in vivo/in vitro assay of tumour immunity.

**DOI:** 10.1038/bjc.1980.11

**Published:** 1980-01

**Authors:** F. Culo, J. M. Yuhas, A. J. Ladman

## Abstract

Multicellular tumour spheroids (MTS) from 4 mouse tumours (Line 1 lung carcinoma; a fibrosarcoma, FSA; a mammary carcinoma, MCa-11; and SV40-transformed fibroblasts, SV-A31) WEre injected into the abdominal cavity of normal, immunized or tumour-bearing syngeneic mice, recovered after 4-48 h, and their growth measured in vitro for 7-16 days. Both normal and immunized mice inhibited MTS growth, but there was no correlation between the two types of inhibition, suggesting that different immunological processes were involved. For example, the greatest inhibition by normal mice was seen for the weakly immunogenic MCa-11, and the highly immunogenic tumour, SV-A31, was only moderately inhibited. However, the summed inhibition of MTS growth in normal and sensitized hosts corresponded to the behaviour of tumours as s.c. transplants; i.e., was inversely related to the malignancy of the same tumours. The inhibition of MTS by mice bearing identical early tumours (FSA or MCa-11) was comparable to that in immunized mice. Histological sections of SV-A31 MTS in normal or immunized hosts revealed the infiltration of MTS by various types of host cells, mostly polymorphonuclears, macrophages and lymphocytes.


*Br. J. Cancer* (1980) **41**, 100

### MULTICELLULAR TUMOUR SPHEROIDS: A MODEL FOR COMBINED *IN VIVO/IN VITRO* ASSAY OF TUMOUR IMMUNITY

F. ČULO,*[3] J. M. YUHAS[1,3] AND A. J. LADMAN[2]

*From the [3]Cancer Research and Treatment Center, [1]Department of Radiology and [2]Department of Anatomy, University of New Mexico, Albuquerque, New Mexico* 87131, *USA*

Received 6 January 1979 Accepted 5 September 1979

**Summary.—Multicellular tumour spheroids (MTS) from 4 mouse tumours (Line 1 lung carcinoma; a fibrosarcoma, FSA; a mammary carcinoma, MCa-11; and SV40-transformed fibroblasts, SV-A31) were injected into the abdominal cavity of normal, immunized or tumour-bearing syngeneic mice, recovered after 4–48 h, and their growth measured *in vitro* for 7–16 days. Both normal and immunized mice inhibited MTS growth, but there was no correlation between the two types of inhibition, suggesting that different immunological processes were involved. For example, the greatest inhibition by normal mice was seen for the weakly immunogenic MCa-11, and the highly immunogenic tumour, SV-A31, was only moderately inhibited. However, the summed inhibition of MTS growth in normal and sensitized hosts corresponded to the behaviour of tumours as s.c. transplants; i.e., was inversely related to the malignancy of the same tumours. The inhibition of MTS by mice bearing identical early tumours (FSA or MCa-11) was comparable to that in immunized mice. Histological sections of SV-A31 MTS in normal or immunized hosts revealed the infiltration of MTS by various types of host cells, mostly polymorphonuclears, macrophages and lymphocytes.**

ANALYSIS of immunological anti-tumour reactions can be conducted either *in vivo* or *in vitro*, each approach having its own advantages and disadvantages. *In vivo* studies are relevant to the complex interactions which must occur with primary tumours, but precise quantitation and isolation of variables is lost. *In vitro* studies, on the other hand, provide precise quantitation, but it is difficult to relate the resultant data to the *in vivo* situation, owing to the large differences in the nature of the target–effector contact. Ideally, a system for studying anti-tumour reactions would include a simulation of the *in vivo* target-effector interaction, followed by a precise *in vitro* analysis.

A potentially useful target for such a method, multicellular tumour spheroids, or MTS, has existed for a number of years. These MTS are growing aggregates of tumour cells, which may be stable enough to permit transfer to appropriate hosts (into the abdominal cavity) followed by harvest and *in vitro* analysis. Furthermore, MTS represent target tumour tissue which has many physical and physiological characteristics in common with a solid tumour *in vivo*. These include cell-to-cell contacts (Sutherland & Durand, 1976), presence of chronically hypoxic cell populations (Sutherland & Durand, 1976; Yuhas *et al.*, 1977; Yuhas & Li, 1978) and cell-cycle times that range from exponential monolayer rates to essentially non-dividing (Sutherland & Durand, 1976). Since antigen expression is dependent on cell-cycle stage (Fenyö *et al.*, 1973), it is

Reprint requests to: Filip Čulo, M.D., Ph.D., Research Scientist, Cancer Research and Treatment Center, 900 Camino de Salud, N.E. Albuquerque, New Mexico 87131.

* On leave of absence from the Department of Physiology, Faculty of Medicine, University of Zagreb, Zagreb, Yugoslavia.

probable that its pattern in MTS would be similar to that for tumours *in vivo*. Furthermore, since live cells encompass several layers in MTS (as in solid tumour *in vivo*), the immunological effectors probably need to penetrate into the solid tumour mass in order to exert the maximum anti-tumour effect. This further implicates the question of how the local, microenvironmental factor may influence immunological reaction, which is largely unknown. Many of these relations are absent when the dissociated cells are considered as a tumour target.

In spite of the obvious advantages of MTS as a target tumour tissue, it has not been used in tumour immunology for a long time. In a few recently reported experiments, MTS was used for *in vitro* (Sutherland *et al.*, 1977) or *in vivo* (MacDonald & Howell, 1978; MacDonald *et al.*, 1978) study of anti-tumour reaction in alloimmune mice. Unfortunately, in these and the other experiments, the methodology of producing of MTS was cumbersome, and only 2 tumour lines, from different species, were available in this form (Sutherland *et al.*, 1971, Sutherland & Durand, 1976). Clearly, this would limit the studies to the analysis of a single tumour in a syngeneic host or 2 tumours in different hosts. Indeed, in reported experiments (Sutherland *et al.*, 1977; MacDonald & Howell, 1978; MacDonald *et al.*, 1978) the response of allogeneic mice to only one tumour was studied.

More recently, however, a simple method for producing MTS has been developed in our laboratory, which has allowed the study of more than 30 different tumours growing as MTS (Yuhas *et al.*, 1977, 1978; Yuhas & Li, 1978). Furthermore, our method allows the growth of MTS in stationary volumes of liquid medium as small as 1 ml and, contrary to those grown in spinner bottles (Sutherland *et al.*, 1971; Sutherland & Durand, 1976), these MTS can be easily handled and assayed for the growth *in vitro*. Since it is now possible to study and compare many tumours, we considered it worth while to pursue the potential of the system, and we present below the results of our initial studies of 4 MTS lines exposed to immune effectors *in vivo* and assayed *in vitro*.

### MATERIALS AND METHODS

*Animals*.—BALB/cJ and C3H/HeJ female mice were obtained from the Jackson Laboratory (Bar Harbor, Maine). The animals were maintained in standard laboratory conditions and were used in experiments at age 3–5 months.

*Tumours*.—Of the mouse tumours used, 3 are of BALB/c origin: a spontaneous alveolar-cell carcinoma (Line 1), a radiation-induced mammary carcinoma (MCa-11) and BALB/c 3T3 fibroblasts (Clone A31) transformed with SV40 virus (SV-A31). The 4th tumour is a fibrosarcoma (FSA) induced by methylcholanthrene in C3H mice. Further details about the origin and history of these tumours has been provided elsewhere (Yuhas *et al.*, 1977). All lines were grown *in vitro* in EBME supplemented with 10% FCS, 50 u/ml of penicillin and 50 $\mu$g/ml of streptomycin (Gibco, Los Angeles, California). Cultures were maintained at 37°C and 100% relative humidity, with the gaseous phase consisting of 5% $CO_2$ in air. Details about growth rate of these tumours *in vitro* as monolayers or as MTS are presented elsewhere (Yuhas & Li, 1978).

The behaviour of these tumours *in vivo* varies widely. Table I shows the pertinent characteristics of each of the 4 tumours studied. At one extreme is the highly malignant, weakly immunogenic Line 1 lung carcinoma, and at the other the strongly immunogenic, low-malignancy SV-A31 fibrosarcoma.

*Immunization*.—With all tumours immunization was performed in syngeneic mice, and with Line 1 also in allogeneic mice. Various sources of cells were used for immunization: monolayers, spheroids and suspensions obtained from *in vivo* tumours. Mice immunized to FSA or MCa-11 were given 3 weekly injections of 1–2 $\times 10^7$ X-irradiated (10,000 rad) tumour cells. These mice were able to reject $10^6$ or more similar live tumour cells. The same procedure was used for immunization of syngeneic mice (BALB/c) to Line 1, except 6 injections of X-irradiated cells were given, and challenge consisted of $10^4$ cells. Only mice which rejected this dose of Line 1 cells (about 80%) within 25–30 days of challenge were considered immune and used in experiments. None of the normal mice rejected the same dose of tumour cells. Allogeneic (C3H) mice were given 3–4 s.c. or i.p. injections of $10^7$ live Line 1 cells, the interval between the first and second injection being 15–25 days, and 1 week between further injections. For immunization to SV-A31 tumour 3 injections of $1\text{–}5 \times 10^6$ live cells were given s.c. at 10–25-day intervals.

All mice, except BALB/c immunized to Line 1 (see above), were used in experiments 15–25 days after the last immunization.

*MTS production.*—Between $10^5$ and $10^6$ tumour cells, harvested from monolayers, were plated in 10 ml of complete media into 100mm Petri dishes, the bottoms of which had been coated with 0·75% agar (Difco, Kalamazoo, Michigan) in complete medium (Yuhas *et al.*, 1977). Within 1–2 weeks MTS ranging in size from 200 to 400 $\mu$m had developed. These MTS are not embedded or attached to the agar-base, but are freely moveable and readily selected and handled.

*Injection and recovery of MTS.*—Approximately 50 spheroids, measuring 250–300 $\mu$m were injected aseptically in 0·5 ml of media into the abdominal cavity of the mouse. Glass 0·25 ml syringes (Becton-Dickinson, Ruthford, New Jersey), which do not retain MTS in the neck, and metal 20-gauge needles were used for injection. Four to 48 h later, mice were killed, injected with 3 ml media and shaken. Fifty units of heparin were added per ml of washing medium. The abdominal cavity was opened and the spheroids removed with a Pasteur pipette, placed in a Petri dish, and washed twice more in fresh medium. An average of 15–30 MTS were recovered from each mouse. Usually, fewer MTS were recovered from immunized than from normal mice. A small percentage of MTS (mostly SV-A31 MTS) lost their spheroid shape during the incubation *in vivo* and appeared either elongated (elliptic) or flat at the time of recovery. The flat MTS were discarded, and the rest of MTS were selected at random (without the use of microscope) for the growth assay. The elongated MTS were sized by calculating the average of two perpendicular diameters. The percentage of MTS which lost spherical shape was proportional to the strength of the immune reaction and our data therefore tend to underestimate the effect of immunization.

*Growth-inhibition assay.*—Ten to 12 MTS were placed individually in agar-based 16mm (24-well) plates, along with 1 ml of medium. The medium was changed twice weekly. They were sized every second day for 7–16 days with a dissecting microscope. The MTS of tumours in these experiments differ considerably in their growth rate (Yuhas & Li, 1978). Therefore, for the sake of comparison, growth-inhibition data for all tumours were calculated when uninjected MTS (media controls) grew to $\sim$400 $\mu$m; for Line 1 and SV-A31 after 4–6 days (growth range 74–85 $\mu$m and 75–87 $\mu$m per day, respectively), for FSA after 8–10 days (growth range 40–51 $\mu$m/day) and for MCa-11 after 14–16 days (growth range 24–28 $\mu$m/day). A certain percentage of Line 1, SV-A31 and FSA MTS (mainly those recovered from immunized mice) completely degenerated during the observation period. Those MTS were not included in growth curves, but their incidence is shown separately. For slow-growing MCa-11 MTS, the observation period (15–16 days) was sometimes too short to observe the complete degeneration of MTS. With that tumour, the MTS which progressively decreased in size and on Day 15 or 16 measured less than half of the original size, were taken as regressed and were excluded from growth curves. Later experiments, in which the growth of these MTS was followed longer, showed that most of such MTS actually regressed completely.

*Colony-forming efficiency (CFE).*—MTS recovered from groups of 2–3 mice were pooled and sized. They were then divided in 2 groups (10–12 MTS in each) of exactly similar sizes. One group was dissociated and plated immediately and the other after growth *in vitro* for 3 days. Each group of MTS was dissociated by incubating them in 0·5 ml of 0·25% Trypsin-EDTA (Gibco) and by occasional aspiration with a 1ml syringe, using a 25-gauge needle. Cells were counted in a haemacytometer, and 400 tumour cells were seeded in 100 mm tissue-culture Petri dishes in 10 ml complete media. The lymphoid cells infiltrating MTS were excluded from counts by their difference in size and morphology. The number of lymphoid cells recovered from MTS was probably too low to produce any significant inhibition of colony growth after the plating (the ratio to tumour cells was at most

1:3). After 9 days of incubation (37° and 5% $CO_2$) the plates were washed, stained with 0·75% crystal violet and colonies scored macroscopically.

*Cyclophosphamide* (*CY*).—Cytoxan (Mead Johnson, Evansville, Indiana) was dissolved in water, and injected i.p. (250 mg/kg) within 1 h.

*Histology*.—MTS were processed for morphological observation by methods which have appeared earlier (Ladman & Yuhas, 1977; Yuhas *et al.*, 1977; Ladman *et al.*, 1979). Sections of 3–6 MTS from identified groups, cut 1–2 $\mu$m in thickness, stained with Toludine Blue O were examined by light microscopy to identify the cells associated with the MTS. In doubtful preparations, identification was checked in thin sections by transmission electron microscopy.

### RESULTS

### *Growth of MTS exposed to normal or sensitized host*

Fig. 1 shows the growth curves for 4 types of MTS from the 4 tumour lines after exposure for 24 h in the peritoneal cavity of normal or immunized syngeneic hosts. For FSA and MCa-11 tumours growth curves are also included of MTS exposed to mice bearing corresponding tumours. MTS which, after the transfer *in vitro*, regressed (see Materials and Methods) are

FIG. 1.—Growth *in vitro* of MTS of 4 tumour lines exposed for 24 h in the peritoneal cavity of normal, immunized, or tumour-bearing mice (mean ± s.e. of MTS recovered from 2–4 mice per group). Numbers on the curves indicate the fraction of MTS which regressed. △, medium controls; ○, normal mice; ●, immunized mice; ■, tumour-bearing mice. Full lines, syngeneic mice; dashes, allogeneic mice. Tumour-bearing mice were injected s.c. with $10^6$ (FSA) or $3 \times 10^6$ (MCa-11) cells 19 or 41 days respectively before the assay (tumour diameters 5–7 mm).

excluded from the growth curves. Their incidences are indicated separately in Fig. 1.

As can be seen, the degree of inhibition of the 4 tumours in both normal and immunized mice differed considerably. The growth pattern of Line 1 recovered from normal syngeneic mice did not differ from that of uninjected MTS (medium controls). The effect of immunization in syngeneic mice was very slight—there was reduction in the growth of only 30–50 $\mu$m in comparison to normal mice. Exposure of the same MTS to allogeneic immunized mice showed that Line 1 is susceptible to the immune attack (Fig. 1). The MTS recovered from these mice decreased in size initially and then resumed growth after a delay of 5–6 days. The growth of Line 1 recovered from normal allogeneic mice was almost identical to that from syngeneic counterparts, and is therefore not shown.

With another tumour, SV-A31, the effect of immunization in syngeneic mice was much more pronounced. The growth curve of MTS recovered from immunized mice resembled that of Line 1 MTS exposed to allogeneic immunized mice: an initial decrease in size and the resumption of growth after 4–5 days. The growth of SV-A31 MTS recovered from normal mice was also significantly inhibited. There was a 1–2-day delay in growth of these MTS in comparison with medium controls.

Essentially, similar effects to SV-A31 tumour were seen with FSA tumour, both in normal and immunized mice. However, the effect of immunization appears less pronounced with FSA. With MCa-11 tumour, a very high degree of growth inhibition was seen in normal mice. MTS exposed to these mice slightly decreased in size initially, and resumed growth after 5–6 days. The effect of immunization was detectable, though not very pronounced. It manifested in a slightly larger initial drop in size and delayed resumption of growth. Mice bearing early FSA and MCa-11 tumours (diameter 5–7 mm) also inhibited significantly the growth of corresponding MTS. In comparison to immunized mice, the inhibition by tumour-bearing mice was somewhat weaker for FSA, and stronger for MCa-11.

Fig. 2.—Influence of CY on MTS growth inhibition by normal animals. △, medium controls; ○, normal animals; ●, normal animals given CY (250 mg/kg) 1 day before MTS injection. Average growth of MTS recovered from 2 animals per group.

Inhibition of growth of SV-A31, FSA and MCa-11 MTS by normal mice could be due either to natural resistance of mice to these tumours, or to experimental artefact (*i.e.*, caused by experimental manipu-

FIG. 3.—Inhibition of MTS of 4 tumours by normal or immunized mice as a function of exposure time. Data for all tumours are taken when medium controls had increased in size to ~400 μm. MTS recovered from 1–4 mice per point: ○, Line 1; ×, MCa-11; △, SV-A31; □, FSA. *A*, normal mice compared to medium controls; *B*, immunized mice compared to normal mice; *C*, immunized mice compared to medium controls. Full lines, syngeneic mice; dashes, allogeneic mice.

lation). To check this, MTS of these tumours were injected either into normal mice or mice given CY (250 mg/kg i.p.) 24 h before. Fig. 2 shows that CY pretreatment almost completely abolished the inhibition of FSA MTS, and to a significant extent also that of MCa-11. This effect was repeatedly observed with MCa-11. Similar results were obtained with SV-A31 (data not shown). Thus the inhibition of MTS by normal mice is due to natural resistance of animals to particular tumours.

Figure 3 shows the inhibition of growth of 4 tumours by normal and immunized mice as a function of exposure time. The differences in size between MTS exposed to normal mice and medium controls (Fig. 3A), and between those exposed to immune and normal mice (Fig. 3B) are shown. In Fig. 3C, the inhibition by normal and immune mice is summarized. Data for different tumours are comparable, since they were taken at equivalent times after MTS transfer *in vitro* (see Materials and Methods). Data on MTS which regressed were not taken into account; their inclusion, however, would not change the overall picture.

The highest degree of inhibition by normal mice was seen with MCa-11. The inhibition of SV-A31 and FSA by normal mice was half or less that of MCa-11. With both tumours it was proportional to the exposure time and, with SV-A31, the inhibition could be detected even after 4 h exposure. There was slight, if any, inhibition of Line 1 in normal syngeneic or allogeneic mice at any exposure time.

In immunized syngeneic mice the highest degree of inhibition, at all exposure times, was found with SV-A31. Significant inhibition of growth of this tumour could be detected after 4 h exposure (more than 100 μm). Inhibition of FSA MTS by immunized mice was about half as strong as with SV-A31. A much smaller effect of immunization was seen with MCa-11 and Line 1. With the latter tumour, very slight inhibition could be detected even after exposure for 16 h or more.

All these tumours were also exposed to normal and immunized host for 48 h (data not shown). With MCa-11 and FSA the effect of immunization changed little from 24 h exposure, owing to the large increase in inhibition by normal mice. SV-A31 MTS could not be recovered from immunized mice—they were already destroyed. Inhibition of Line 1 MTS by immunized mice increased proportionally, but was not detectable in normal mice.

Inhibition of tumours by normal mice (natural resistance) and induced immune response probably both play a role in the resistance of host to the tumour. The overall inhibition of MTS growth in immunized mice by comparison with medium controls (Fig. 3C) is probably the sum of the interaction of both natural and induced immunity. The degree of inhibition of different tumours, as presented in Fig. 3C, correspond fairly well to the behaviour of the subcutaneous transplants of the same tumours (Table I), being highest with the least malignant tumour (SV-A31), lowest with most malignant tumour (Line 1) and intermediate with moderately malignant tumours (FSA and MCa-11). The degree of inhibition of SV-A31 in syngeneic mice was comparable to that of Line 1 in allogeneic mice. Similar correlations to *in vivo* malignancy of tumours could be found in the incidence of the regression of MTS of different tumours (Fig. 1). It was the highest with SV-A31 (more than 50% regressions with 24h exposure) and the lowest (nil) with Line 1.

*Specificity of growth inhibition in immunized mice*

This was tested for SV-A31, Line 1 and MCa-11 MTS in criss-cross pattern in syngeneic (BALB/c) mice. A batch of mice was immunized to a particular tumour, and all 3 types of MTS were given to the mice from the same batch. At the same time, MTS were given to the unimmunized mice as a control. All MTS were recovered after 24 h, except for Line 1, where, to obtain a measurable inhibition, they were recovered after 48 h. Table II shows the net differences in size of MTS exposed to immunized and normal mice. For different tumours, values are taken at equivalent times after the transfer (see Materials and Methods). As can be seen, immunization to Line 1 significantly inhibited MCa-11 and SV-A31 MTS, but to a lesser extent. Immunization to SV-A31 or MCa-11 was specific; only MTS of respective tumours were significantly inhibited

In another experiment, the specificity of growth inhibition of FSA MTS in C3H mice was tested. Since MTS of other tumours of C3H origin were not available, cross-reactivity in C3H mice was tested against allogeneic (Line 1) tumour. As

TABLE I.—*Characteristics of 4 tumours used in the present study*

| Tumour | Host | Aetiology | Minimum cell dose for transplant* | Immunogenicity† | Spontaneous metastasis‡ | S.c. growth rate (mm/day) |
|---|---|---|---|---|---|---|
| Line 1 | BALB/c | Spontaneous | $\sim 10^2$ | Weak | 100% | Rapid (0·56 ± 0·07) |
| SV-A31 | BALB/c | Virus-induced | $3\text{–}10 \times 10^7$ | Strong | None detected | Very rapid (1–1·3) |
| MCa-11 | BALB/c | Radiation-induced | $1\text{–}5 \times 10^6$ | Weak to moderate | Rare | Slow (0·23 ± 0·04) |
| FSA | C3H | Chemically-induced | $1\text{–}5 \times 10^6$ | Moderate | Extremely rare | Rapid (0·56 ± 0·07) |

* Minimum dose of monolayer-derived cells to produce 100% takes of progressively growing tumours.

† Based on ability of dead tumour-cell immunization to increase the live-cell dose for successful transplantation.

‡ Frequency of spontaneous metastatic spread to the lungs from s.c. transplants.

TABLE II.—*Specificity of growth inhibition of MTS in BALB/c mice*

| Mice immunized to | Expt | Growth inhibition* Target MTS‡ Line 1 | MCa-11 | SV-A31 |
|---|---|---|---|---|
| Line 1 | 1 | − 99 ± 13† | − 65 ± 26† | − 36 ± 20 |
| | 2 | − 136 ± 41† | − 29 ± 35 | − 51 ± 18† |
| | Mean | − 117·5 | − 47 | − 43·5 |
| MCa-11 | 1 | − 12 ± 35 | − 130 ± 30† | |
| | 2 | 6 ± 21 | − 100 ± 36† | |
| | 3 | | − 92 ± 38† | − 17 ± 28 |
| | 4 | | − 106 ± 29† | − 8 ± 16 |
| | Mean | − 3 | − 107 | − 12·5 |
| SV-A31 | 1 | − 8 ± 17 | − 27 ± 36 | − 319 ± 35† |
| | 2 | 12 ± 18 | − 15 ± 20 | − 350 ± 80† |
| | Mean | 2 | − 21 | − 334·5 |

* The differences in size (μm ± s.e.) between MTS exposed to immunized and normal mice (n ≮ 18; 1–2 per group). Negative sign denotes slower growth in immunized group (inhibition). Data are compared when uninjected MTS of all 3 tumours grew to ~400 μm.

† Significant at $P < 0{\cdot}05$ (Student's *t* test).

‡ Line 1 MTS were exposed to normal or immunized host for 48 h; MCa-11 and SV-A31 for 24 h.

TABLE III.—*Specificity of growth inhibition of MTS in C3H mice*

| Mice Immunized to | Expt | Growth inhibition* target MTS† Line 1 | FSA |
|---|---|---|---|
| Line 1 | 1 | − 413 ± 35‡ | − 72 ± 18‡ |
| | 2 | − 401 ± 22‡ | − 30 ± 39 |
| | 3 | − 423 ± 15‡ | − 92 ± 21‡ |
| | Mean | − 412 | − 65 |
| FSA | 1 | 8 ± 18 | − 176 ± 16‡ |
| | 2 | − 26 ± 15 | − 118 ± 46‡ |
| | Mean | − 9 | − 147 |

* See footnotes to Table II.

† Exposed for 24 h to normal or immunized host.

‡ Significant at $P < 0{\cdot}05$ (Student's *t* test).

Table III shows, immunization to a syngeneic tumour (FSA) had no influence on the growth of an allogeneic tumour. However, immunization to Line 1 sometimes significantly inhibited also FSA MTS, but far less than Line 1.

*Colony-forming efficiency of MTS exposed to normal or immunized host*

In order to see whether the growth inhibition assay used in our experiments correlated with other *in vitro* assays which measure cell survival and reproduction, the colony-forming efficiency (CFE) of *in vivo* exposed MTS was investigated. Line 1 and SV-A31 MTS exposed to syngeneic normal or immunized host were dissociated and plated either immediately after recovery (Day 0) or after growth *in vitro* for 3 days. In Table IV, the results are expressed as relative CFE (number of colonies per 400 plated cells) and as a number of clonogenic units (CU) per MTS.

At Day 0, the CFE of Line 1 MTS recovered from normal mice was similar to medium controls, and of those recovered from immunized mice slightly reduced (22%). The number of CU was slightly reduced in normal group, and much more so in immunized group (2·4 times less CU than in medium controls). The greater decrease in number of CU per MTS is due to lower cell counts in MTS (of same size) exposed to mice than in medium controls. After 3 days' growth *in vitro* of Line 1 MTS, almost all differences between groups disappeared. Still, there was a slight reduction in the number of CU per MTS in the immunized group, which correspond to a slightly slower growth of the same MTS.

In comparison to Line 1 MTS, at Day 0, both the reduction of CFE and CU due to

TABLE IV.—*Colony forming efficiency of MTS exposed for 24 h to normal or immunized mice*

| Tumour | Host | Time elapsed between recovery of MTS and assay (days)* 0: Colonies/400 cells (%) | 0: Colonies/MTS (×10³) | 3: Colonies/400 cells (%) | 3: Colonies/MTS (×10³) | Growth of MTS (μm) |
|---|---|---|---|---|---|---|
| Line 1 | | 79·2 ± 5·1 (19·8) | 3·6 | 71·7 ± 2·2 (17·9) | 6·4 | 238 ± 15 |
| | Normal | 85·3 ± 6·9 (21·3) | 2·9 | 70·0 ± 2·4 (17·5) | 6·6 | 235 ± 5 |
| | Immunized | 61·8 ± 4·5 (15·5) | 1·5 | 67·7 ± 2·3 (16·9) | 5·4 | 194 ± 9 |
| SV-A31 | | 71·2 ± 1·8 (17·8) | 2·1 | 65·5 ± 1·0 (16·4) | 4·0 | 248 ± 20 |
| | Normal | 61·7 ± 1·8 (15·4) | 1·5 | 62·5 ± 2·5 (15·6) | 3·2 | 202 ± 28 |
| | Immunized | 28·2 ± 2·2 (7·1) | 0·53 | 18·3 ± 1·9 (4·6) | 0·22 | −51 ± 27 |

* MTS were recovered from groups of 2–3 mice, pooled, dissociated and plated either immediately (Day 0) or after growth *in vitro* for 3 days. For each tumour, exactly similar sizes of MTS were used in different treatment groups and also on Day 0 and 3 within the same group (MTS diameter 418 ± 11·3 μm for SV-A31 and 394 ± 6·3 μm for Line 1).

exposure in immunized mice were more pronounced in SV-A31 MTS. Both of these parameters were also significantly reduced in SV-A31 MTS exposed in normal mice. By allowing SV-A31 MTS to grow *in vitro* for 3 days, the differences between MTS exposed to normal mice and medium controls diminished. On the contrary, in immunized group all the differences from medium control or normal mice enlarged. Thus, in comparison to medium controls, the number of CU in immunized group was reduced ~ 4-fold at Day 0 and 18-fold at Day 3. This parallels well the difference in size of MTS in two groups after 3 days' growth *in vitro* (299 μm). It is interesting that in the immunized group the CFE was more reduced and the number of CU smaller at Day 3 than at Day 0. This may mean that delayed (3 days') dissociation of MTS allowed prolonged (intimate) contact of immune effectors and tumour cells, which induced additional killing. However, more data are needed to substantiate this.

### *Histological examinations*

SV-A31 MTS implanted into normal or immune syngeneic mice were examined histologically. Both MTS exposed to the normal and immunized mice were infiltrated by many polymorphonuclear neutrophilic leucocytes, fewer lymphocytes and occasional macrophages. The extent of infiltration by leucocytes in normal animals was similar to that in immune animals. This was confirmed by haemacytometer counts, in which about one third of the cells conformed to leucocyte size and morphology in both normal and immunized mice. The degree of infiltration varied between individual MTS, being much greater in those that had lost their spheroid shape, suggesting that the initial stages of degeneration may have begun. The presence of cells within the MTS, with the morphology of polymorphonuclear neutrophilic leucocytes, lymphocytes and macrophages, was confirmed by electron microscopy.

### DISCUSSION

Although MTS, as any *in vitro* growing tumour tissue, lack some characteristics of tumours *in vivo* (*e.g.* they are not vascularized), they do have many properties (morphological, metabolic and kinetic which closely resemble those of solid tumours (Sutherland & Durand, 1976;

Yuhas *et al.*, 1977; Yuhas & Li, 1978). For example, although they are not vascularized, the proportion of oxygen-dependent dividing cells to undividing cells in MTS seems to be close to that in the *in vivo* growing tumour (Yuhas, 1980). Therefore, MTS may be well suited to the study of immunity to solid tumours. Although it has been shown that MTS are sensitive to *in vitro* attack of alloimmune cells (Sutherland *et al.*, 1977), MTS offer a unique possibility of a combined *in vivo*/*in vitro* study of tumour immunity. Therefore, in our present experiments we tried to devise an assay in which tumour targets (MTS) are exposed to immunological effectors *in vivo*, the effect of which is followed *in vitro*. Such an assay combines the advantages of both *in vivo* and *in vitro* assays; *i.e.*, exposure of tumour cells in the most physiological environment (the animal), followed by quantitative *in vitro* analysis. This approach has also been recently used by others (MacDonald & Howell, 1978; MacDonald *et al.*, 1978), for study of the effector phase of immune reaction to allogeneic tumours. In the present study, immunity of syngeneic immunized or tumour-bearing mice, as well as of allogeneic mice, was tested against several murine tumours.

Various other parameters commonly used in *in vitro* assays, such as colony-forming efficiency (CFE) (Sutherland *et al.*, 1977; MacDonald & Howell, 1978; present experiments) or isotope incorporation (Čulo & Yuhas, unpublished) can be easily used in our system for the detection of the action of immune effectors on MTS. We are using the inhibition of growth of MTS as a parameter of tumour immunity because of its simplicity, and the possibility of following the effect on the same target for a long time. Furthermore, the growth of MTS *in vitro* can be easily related to the situation *in vivo*, progression or regression of solid tumour mass being the observed effect in both cases. Thus, in the present experiments there is a good correlation between the degree of growth inhibition (Fig. 3) or incidence of MTS regression (Table I) and the growth of the same tumours *in vivo*. Also, it seems that the growth pattern of MTS parallels quite well the other more commonly used parameters for measuring cell survival and reproduction *in vitro*. This was demonstrated in the experiment in which CFE and the growth of MTS were measured. Both, the growth inhibition and reduction of CFE in syngeneic mice were more pronounced with highly immunogenic (SV-A31) than with lowly immunogeneic (Line 1) tumour (Table IV). The slight discrepancy between the two assays, appearing when Line 1 MTS recovered from normal mice and medium controls are compared (significant reduction of CU/MTS and practically no inhibition of growth), might indicate that CFE may be more sensitive to damage of tumour cells than is the growth assay. However, this may be only an artefact.

For example, during incubation in a syngeneic host, MTS might, for some reason, swell and increase in size, without a proportional increase in cell count. Naturally, this will reduce the number of clonogenic units relative to medium controls of the same size. Supporting this are the facts that relative CFE (number of colonies per 400 cells) was not reduced, and that the differences from medium controls disappeared after 3 days' culture *in vitro*.

A high degree of tumour immunity could be detected in the Line 1 system in alloimmune mice. Other investigators showed that plating efficiency of MTS exposed to alloimmune mice is greatly reduced, 99% or more reduction of clonogeneic cells was obtained with 48h exposure (MacDonald & Howell, 1978). In our experiments we were unable to recover the MTS (Line 1) from alloimmune mice after 48 h—they were already destroyed. This may be partly due to a lower MTS size used in our experiments than in those mentioned above (250–350 *vs* 700–900 $\mu$m). However, almost 50% Line 1 MTS regressed after 24 h exposure to alloimmune mice, indicating that few if any tumour cells survived.

More interesting, however, was the effect of immune response of syngeneic animals on the growth of MTS. By using 4 tumour lines, it was shown that 3 tumours were inhibited to a high degree by normal animals. Inhibition of some MTS by normal animals might reflect the natural killer activity, which was repeatedly seen in mice both *in vivo* and *in vitro* (reviewed by Herberman & Holden, 1978). As in short-term $^{51}$Cr-release assay, commonly used for detection of natural killer activity *in vitro*, inhibition of MTS growth (SV-A31) could be detected after a 4 h exposure. On the other hand, exposure of a resistant tumour line (Line 1) for 48 h to normal animals produced no measurable anti-tumour effect. The inhibitory effect of normal mice was significantly reduced by their pre-treatment with CY, an agent known to abolish natural killer activity (Oehler & Herberman, 1978). Furthermore, inhibition of SV-A31 MTS was stronger in young than in old animals, and in T-cell-deprived animals than in normal (Čulo & Yuhas, unpublished). Although these observations are in agreement with known facts about natural killer activity (Herberman & Holden, 1978; Shellam, 1977), further study is required before definite conclusions can be reached concerning the relationship of our observations to classical natural killer mechanisms.

The effect of immunization of syngeneic animals to 4 tumours differed widely. It was barely detectable with Line 1, and highly pronounced with SV-A31. By looking at either growth curves or incidence of tumour regressions (Fig. 1) or the quantitative degree of inhibition (Fig. 3) striking similarities could be found between inhibition of SV-A31 MTS by syngeneic immunized mice and Line 1 MTS in allogeneic immunized mice.

The immunity of tumour-bearing animals could also be detected by this assay. The inhibition of MTS growth by mice bearing early FSA or MCa-11 tumours, when compared to corresponding immunized mice, was either somewhat weaker (FSA) or significantly stronger (MCa-11). It appears, therefore, that the MTS growth-inhibition assay might be well suited to study of the concomitant immunity *in vivo*. The advantage of this assay over other *in vivo* assays would be the opportunity for variation of exposure to tumour, use of small number of animals, *etc.*

There was no correlation between susceptibility of tumours to natural immunity effectors and their immunogenicity. Thus MCa-11 was very susceptible to natural effectors and of rather low immunogenicity. Natural resistance to SV-A31was moderate and immunogenicity very high, *etc.* This is most probably related to different effector cells being involved in natural and induced immunity (Herberman & Holden, 1978) and, therefore, to different recognition structures involved in the two processes.

Both natural resistance and induced immunity play varying roles in resistance of animals to tumours *in vivo*. The correlation of the growth inhibition of MTS to the growth or malignancy of tumours *in vivo* was seen only if inhibition of MTS growth in normal and immunized mice was summed (Fig. 4C).

Inhibition of MTS growth *in vivo* seems to be fairly specific in immunized syngeneic mice, at least for 3 out of 4 tumours tested (SV-A31, MCa-11 and FSA). Immunization to Line 1 tumour induced some inhibition of 2 other syngeneic tumours. Similarly, allogeneic mice immunized to Line 1 tumour also inhibited somewhat the syngeneic (FSA) tumour, whereas there was no cross-reactivity in the opposite direction. Inhibition of syngeneic tumours by mice immunized to allogeneic tissue has also been reported by others (Kobayashi *et al.* 1974; Bear *et al.*, 1977), and is thought to be caused by nonspecific immune mechanisms (Gotohda *et al.*, 1976). The undirectional cross-reactivity might, for example, be due to the boosting of natural killer activity by one tumour but not by the other, and thus cross-reactivity in the classical sense (sharing of antigens) might not be involved.

Regarding the mechanism of the inhibition of MTS growth, it is probable that it is mediated by cellular, not humoral mediators, at least in the allogeneic situation. Thus we were unable to show any growth inhibition of Line 1 MTS implanted in a diffusion chamber into alloimmune mice, or exposed *in vitro* to the serum of the same animals (unpublished). Using the $^{51}$Cr-release assay, MacDonald *et al* (1978) showed that in the alloimmune system the cytotoxic cells within MTS are nonadherent T cells. However, in another model, in which the cytotoxicity of immune cells infiltrating the *in-vivo*-implanted sponges previously filled with allogeneic cells was investigated, the non-T allogeneic immune cells showed the highest activity (Roberts & Häyry, 1976; Häyry & Roberts, 1977). In both models, the cells which infiltrated the graft were more cytotoxic per cell than cells outside the graft (*i.e.*, in the peritoneal cavity, spleen or lymph nodes). No significant infiltration of allogeneic MTS by normal host cells was seen (MacDonald *et al.*, 1978). In our experiments, however, where a syngeneic model (SV-A31) was used, the infiltration of cells from normal mice was almost as extensive as from immunized mice. Although the difference may originate from the different models used (allogeneic *vs* syngeneic), it is probably related to the fact that normal mice exerted an anti-tumour activity in our model, but not in model of MacDonald *et al.* (1978).

Interestingly, all kinds of leucocytes (polymorphonuclears, lymphocytes, macrophages and plasma cells) and probably even mast cells could be seen within syngeneic MTS. Similar data were obtained when infiltration by host cells of *in-vivo*-growing tumour was studied (Haskill *et al.*, 1975). The role of these subpopulations of syngeneic lymphoid cells in MTS growth inhibition is at present being investigated in an *in vitro* assay.

This work has been supported financially by Grant No. 5 PO1 CA 14052-05 and Grant No. PO1 CA 16127-03 from the NCI DHEW. The skilful technical assistance of Mr D M Thompson is gratefully acknowledged.

### REFERENCES

Bear, R. H., Roholt, O. A. & Pressman, D. (1977) Syngeneic tumor rejection induced by immunization with normal allogeneic tissues. *Immunol. Commun.*, **6**, 217.

Fenyö, E. M., Peebles, P. T., Wahlström, A., Klein, E. & Cochran, A. J. (1973) Changes in cell surface properties during the *in vivo* growth of Moloney lymphoma. In *Immunological Parameters of Host–Tumor Relationships*, Vol. 2. Ed. D. W. Weiss. New York: Academic Press. p. 35.

Gotohda, E., Kawamura, T., Sendo, F. & 6 others (1976) Effect of combination treatment with cyclophosphamide and nonspecific passive immunization on a transplantable tumor in WKA rats. *Cancer Res.*, **36**, 2119.

Haskill, J. S., Yamamura, Y. & Radov, L. (1975) Host response within solid tumor: Non-thymus-derived specific cytotoxic cells within a murine mammary carcinoma. *Int. J. Cancer*, **16**, 798.

Häyry, P. & Roberts, P. J. (1977) Allograft-infiltrating killer cells. *Transplant. Proc.*, **9**, 691.

Herberman, R. B. & Holden, H. T. (1978) Natural cell-mediated immunity. *Adv. Cancer Res.*, **27**, 305.

Kobayashi, H., Gotohda, E., Kuzumaki, N., Takeichi, N., Hosokawa, M. & Kodama, T. (1974) Reduced transplantability of syngeneic tumors in rats immunized with allogeneic tumors. *Int. J. Cancer*, **13**, 522.

Ladman, A. J., Yuhas, J. M., Li, A. P. & Martinez, A. O. (1979) Ultrastructural observations of three murine tumors grown as multicellular tumor spheroids: Lung carcinoma (Line 1), fibrosarcoma (FSA) and mammary carcinoma (MCa-11). *Cancer Res.* (In press).

Ladman, A. J. & Yuhas, J. M. (1977) Electron microscopic observations of intracisternal A type particles in a transplantable murine alveolar cell carcinoma with remarks on the cellular origin of this tumor. In *Pulmonary Macrophage and Epithelial Cells*. Eds. Saunders, Schneider, Doyle and Ragen. Washington: Batelle Memorial Institute. p. 205.

MacDonald, H. R. & Howell, R. L. (1978) The multicellular spheroid as a model tumor allograft. I. Quantitative assessment of spheroid destruction in alloimmune mice. *Transplantation*, **25**, 136.

MacDonald, H. R., Howell, R. L. & Macfarlane, D. L. (1978) The multicellular spheroid as a model tumor allograft. II. Characterization of spheroid-infiltrating cytotoxic cells. *Transplantation*, **25**, 141.

Oehler, J. R. & Herberman, R. B. (1978) Natural cell-mediated cytotoxicity in rats. III. Effect of immunopharmacologic treatments on natural reactivity and on reactivity augmented by polyinosinic-polycytidilic acid. *Int. J. Cancer*, **21**, 221.

Roberts, P. J. & Häyry, P. (1976) Effector mechanisms in allograft rejection. I. Assembly of "sponge matrix" allografts. *Cell. Immunol.*, **26**, 160.

Shellam, G. R. (1977) Gross-virus-induced lymphoma in the rat. V. Natural cytotoxic cells are non-T-cells. *Int. J. Cancer*, **19**, 225.

SUTHERLAND, R. M. & DURAND, R. E. (1976) Radiation response of multicell spheroid—an *in vitro* tumor model. *Curr. Top. Radiat. Res.*, **2**, 87.

SUTHERLAND, R. M., MCCREDIE, J. A. & INCH, R. W. (1971) Growth of multicell spheroids in tissue culture as a model of nodular carcinoma. *J. Natl Cancer Inst.*, **46**, 113.

SUTHERLAND, R. M., MACDONALD, H. R. & HOWELL, R. L. (1977) Multicellular spheroids: A new model target for *in vitro* studies of immunity to solid tumor allografts. *J. Natl Cancer Inst.*, **58**, 1849.

YUHAS, J. M. (1980) A theoretical comparison of oxygen gradients in solid tumour spheroids. *Cancer Res.* (In press).

YUHAS, J. M. & LI, A. P. Determinants of growth rate of multicellular tumor spheroids (MTS) derived from seven murine tumors. *Cancer Res.*, **38**, 1528.

YUHAS, J. M., LI, A. P., MARTINEZ, A. O. & LADMAN, A. J. (1977) A simplified method for production and growth of multicellular tumor spheroids. *Cancer Res.*, **37**, 3639.

YUHAS, J. M., TARLETON, A. E. & MOLZEN, K. B. (1978) Dormancy and spontaneous recurrence of human breast cancer *in vitro*. *Cancer Res.*, **38**, 2486.